# Detection of Invertebrate Suppressive Soils, and Identification of a Possible Biological Control Agent for *Meloidogyne* Nematodes Using High Resolution Rhizosphere Microbial Community Analysis

**DOI:** 10.3389/fpls.2016.01946

**Published:** 2016-12-26

**Authors:** Nigel L. Bell, Katharine H. Adam, Rhys J. Jones, Richard D. Johnson, Yeukai F. Mtandavari, Gabriela Burch, Vanessa Cave, Catherine Cameron, Paul Maclean, Alison J. Popay, Damien Fleetwood

**Affiliations:** ^1^Soil Biology Team, AgResearch Ltd, Ruakura Research CentreHamilton, New Zealand; ^2^Plant/Fungal Interactions Team, AgResearch Ltd, Grasslands Research CentrePalmerston North, New Zealand; ^3^Bioinformatics and Statistics Team, AgResearch Ltd, Ruakura Research CentreHamilton, New Zealand; ^4^Bioinformatics and Statistics Team, AgResearch Ltd, Lincoln Research CentreChristchurch, New Zealand; ^5^Biotelliga Ltd, Institute for Innovation in BiotechnologyAuckland, New Zealand

**Keywords:** *Trifolium repens*, white clover, rhizosphere, grass grub, mānuka beetle, next generation sequencing, Mi-Seq, biological control

## Abstract

White clover (*Trifolium repens*) is the key legume component of New Zealand pastoral agriculture due to the high quality feed and nitrogen inputs it provides. Invertebrate pests constrain white clover growth and this study investigated rhizosphere-associated fungal controls for two of these pests and attempts to disentangle the underpinning mechanisms. The degree of suppressiveness of 10 soils, in a latitudinal gradient down New Zealand, to added *Meloidogyne hapla* and *Costelytra zealandica* scarab larvae was measured in untreated soil. Most of the soils showed no suppressive activity against these pests but two showed activity against *M. hapla* and two against *C. zealandica*. Rhizosphere fungi responsible for pest suppressive responses were elucidated via next-generation sequencing. In the *M. hapla*-suppressive soils nematode-trapping Orbiliomycetes fungi were present in significantly greater abundance than non-suppressive soils and their abundance increased further with addition of *M. hapla*. A comparison of plant growth and the rhizosphere fungal community between untreated and irradiated soil was carried out on 5 of the 10 soils using *Pyronota* as the scarab larvae. Soil irradiation either: reduced (by 60–70%); increased (16×) or made no difference to white clover growth across the five soils tested, illustrating the range of microbial impacts on plant production. In one of the *M. hapla* suppressive soils irradiation resulted in a significant increase in nematode galling suggesting that Orbiliomycetes fungi were indeed responsible for the suppressive effect. Lack of consistent changes in soil macronutrients and pH post-irradiation suggest these were not responsible for plant or invertebrate responses. The use of next generation sequencing in controlled pot trials has allowed identification of a potential biological control organism and bioindicator for *M. hapla* suppression.

## Introduction

New Zealand agriculture is dominated by pastoral grazing systems used to grow animals for their meat, milk, or wool (Statistics New Zealand, 2015). Pastures in these systems largely consist of grass/legume mixtures of various sorts with the majority of mixtures being *Lolium* (mostly *L. perenne*)/*Trifolium* (mostly *T. repens*) (Li et al., 2011). In these mixtures the grass provides the bulk of the grazing ruminant’s diet with the legume supplying high protein forage and nitrogen fixation capability. Pastures occur across a broad range of soil and climate conditions the length and breadth of the country (Cullen et al., 2008) so face a range of biotic and abiotic challenges and interact with a wide range of soil microbes. Of the biotic challenges to pastures, invertebrates, including root-feeders, represent a significant check on plant growth (see Goldson et al., 2015).

Invertebrates with contrasting scales and types of impact on *T. repens* include scarab beetles (such as *Costelytra zealandica* and *Pyronota* spp.) and nematodes, such as *Meloidogyne hapla.* Scarab larvae consume plant root tissue (Kain and Atkinson, 1977), reducing root mass and impinging on root function which can result in plant death when they occur at sufficiently high populations densities (East et al., 1980). *M. hapla* juveniles invade root tissue immediately behind the root growing point and establish a permanent feeding site by means of induction of giant cells which are created via plant growth regulator stimulation of the plant tissue (Moens et al., 2010). The resulting galls (or “knots,” which give them the common name root-knot nematodes) impair root function but usually only result in plant death if accompanied by other stressors such as low soil nutrient status or water deficit.

The rhizosphere is the area of soil directly in contact with plant roots or their secretions and as such is the site of intense interactions between the plant and soil microbes. This zone is also the first point of contact between plants and invertebrate root-feeders, with plant secretions being used as food signals for root-feeders (see van Dam and Bouwmeester, 2016). It is therefore likely that at least some of the microbes in this zone would be adapted to utilizing soil invertebrates as a food resource (either as closely associated root residents or as recruits in response to plant damage, as occurs in disease responses (see Berendsen et al., 2012), making the rhizosphere an important area to target in the search for biological control (biocontrol) agents against pest invertebrates. Traditionally, invertebrate pathology studies have uncovered microbial biocontrol agents by examining diseased individuals and this has been successful for bacteria against *C. zealandica* in New Zealand (Grimont et al., 1988), and for some *Meloidogyne* species overseas (see Davies, 2009). A rhizosphere-based approach to detecting biocontrol agents would, alternatively, need to consider which microbes increase in abundance in response to herbivore feeding on, or invasion of, roots. Those microbes responding most strongly would then be good candidates for biocontrol agents that are also rhizosphere-competent, an important consideration for persistence of agents in the soil. Along with potential biocontrol agents, a rhizosphere-based approach may identify bioindicators of pest suppression. Such bioindicators could be used to quantify the need, or not, for control measures to be implemented as part of operations such as pasture renewal or crop rotations (e.g., Ophel-Keller et al., 2008).

A culture-dependant method for a rhizosphere-based approach to detecting organisms responsible for soil pest suppression would clearly underestimate the diversity of potential biocontrols and bioindicators. Culture dependant techniques also require either: some prior knowledge of an existing pest suppression mechanism; or that a wide variety of culturing isolations be employed to find any causative mechanism. Culture-independent techniques, by contrast, allow for enumeration of the majority of microbes in soil and would provide clearer evidence of potentially useful biocontrols or bioindicators. Techniques such as high-throughput sequencing are rapidly developing and have recently moved from 454 pyrosequencing to the Illumina Mi-Seq platform (Derakhshani et al., 2016) which can generate up to 150-bp sequencing reads with a total throughput of 1.5–2 Gb per run. Such technologies have facilitated analysis of environmentally derived samples from a variety of ecosystems, including soil, and can measure very small changes in community structure across different samples (Schmidt et al., 2013).

The focus of this study was the below-ground biotic challenge to *T. repens* presented by root feeding invertebrates and the changes that this challenge evokes within the rhizospheric fungal microbiota. We have utilized next generation sequencing to explore the fungal microbiota present in the rhizosphere of *T. repens* across a range of New Zealand soils and in the face of challenges from these invertebrate pests. We hypothesize that near-isogenic *T. repens* plant performance will differ in different soils from across New Zealand and that this will be linked to interactions between components of the rhizosphere microflora and soil dwelling root pests. A substantial comparison of the high resolution microbial communities will form the basis of a separate paper.

## Materials and Methods

### Pot Trials

**FIGURE 1:**
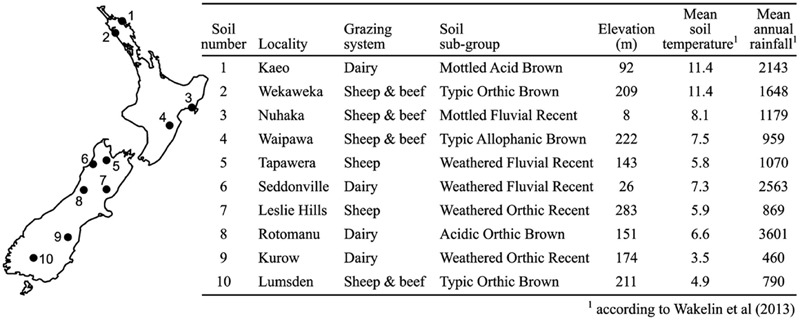
**Location, management, climate, and characteristics of soils used in this study**.

Soil samples were collected from 10 sites previously characterized by Wakelin et al. (2013) that represented a wide range of geographical locations and soil characteristics, primarily pH and nitrogen levels (**Figure 1**). In addition, half the sites were on brown soil type and half on recent soils, while four sites were dairy farms and six were sheep and beef farms. From each site 200 mm × 200 mm × 200 mm cubes of soil were excised between January and February 2014 or August and September 2014 for Experiments 1 and 2, respectively. The top 15 mm of plant matter and soil was removed from each cube and the remainder stored in sealed buckets at 4°C. Collection and manipulation of soil was performed with gloves and implements rubbed with 70% ethanol to sanitize.

Soil was passed through a 4 mm sieve and mixed by hand to homogenize. Nematodes were extracted and counted from 100 g of each soil using the method of Bell and Watson (2001). The extraction ran for 72 h, after which the resulting nematode suspension was reduced to a final volume of 20 ml by a combination of beaker settling and aspiration. The total number of nematodes and plant parasitic nematodes were counted in a Doncaster dish (Doncaster, 1962) under a stereo-microscope at 40–80× magnification; plant parasitic nematodes were identified to genera based on the keys of Siddiqi (2000) for Tylenchida and Bongers (1994) for other groups. To allow for valid comparison across treatments, results are presented as nematodes per 100 g of dry soil. Further subsamples from each soil were sent to Hills Laboratory (Hamilton) for soil nutrient analysis [pH, Olsen P, Sulfate S, K, Ca, Mg, Na, total N and cation exchange capacity (CEC), see http://www.hill-laboratories.com/file/fileid/15530 for methods].

Initial moisture of each soil was determined from three 10 g samples oven dried at 80°C for 24 h. Water holding capacity (WHC) was determined by adding 180 g of each soil into small pots (50 mm × 50 mm × 120 mm), weighing the combination of pots with soil, soaking for 24 h, draining for 72 h and re-weighing. The 100% WHC of the system was calculated as the total weight of water held in the system (including initial soil moisture).

**FIGURE 2:**
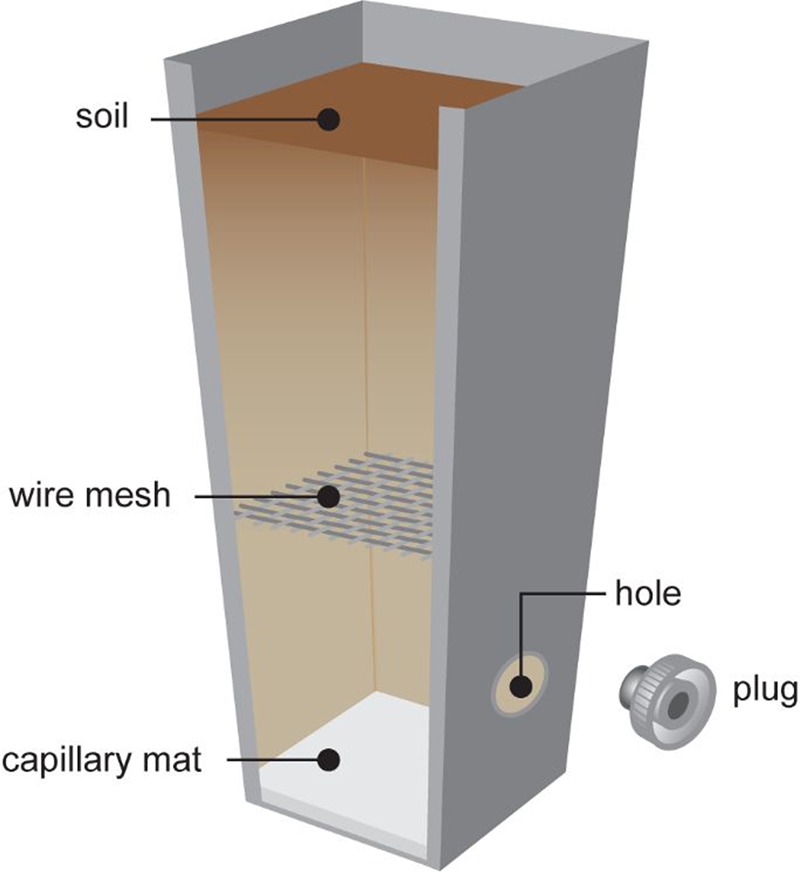
**Plant pot with wire mesh as scarab larval excluder and plug for inoculating larvae into pot**.

Pots were filled with 180–200 g soil per pot, depending on soil, with capillary mat inserted at the base of the pots to assist with water and soil retention. Pots were designated to one of three treatments: clover-only control; clover with *M. hapla* added; or clover with root-feeding scarab larvae added. For the larvae-inoculated pots a plugged hole and wire mesh were incorporated into the pot (**Figure 2**) to allow for introduction of the larva and to prevent larvae consuming the entire clover root system, respectively. Pots were arranged in a randomized split plot design with soils allocated to the whole-plots and treatments to the subplots.

Near-isogenic white clover seed (S9) was obtained from a breeding population developed within AgResearch derived from cv. Crau. A near-isogenic seedline was selected in an attempt to reduce between-plant variability in growth and reaction to imposed treatments. Seeds were scarified by abrasion with ‘wet and dry’ sandpaper then surface sterilized by soaking for 15 min in 10N H_2_SO_4_ containing a drop of Tween 80, then washing five times in sterile milliQ water. Seeds were sprouted on moist sterile filter paper at 20°C for 3 days prior to planting into pots. A single plant was sown into each pot. Aseptic techniques were used during planting to prevent cross contamination between soils. Plants were grown at isothermal 20°C under high pressure sodium vapor lamp lighting on a 16 h light and 8 h dark cycle. *M. hapla* and scarab larvae were added 5 weeks after sowing clover plants into pots.

MilliQ water was initially added to each pot to bring moisture levels up to 70% WHC. Once a week each pot was reweighed and returned to 70% WHC. Between each weekly weighing pots were checked daily and any that appeared dry were watered with 3–5 ml. Following inoculation of the plants with *M. hapla* or scarab larvae watering was reduced to 60% WHC in Experiment 1 but maintained at 70% WHC in Experiment 2.

*Meloidogyne hapla* stock populations [confirmed as such by ITS sequencing (555 bp sequence 99% match to *M. hapla* (GenBank reference: LC030360), query coverage 100%, see Rohan et al., 2016 for PCR methodology)] were established and maintained on tomato plants (*Solanum lycopersicum* cv. ‘Rutgers’) under glass house conditions. After 4 months galled tomato roots were excised from the tomato plants, and nematode eggs were collected following Aalders et al. (2009). The resulting inoculum consisted of 1089 (± 31 SEM) eggs and 20 (± 6) J2 juvenile *M. hapla* per ml with 346 (± 38) of the eggs being embryonated. A 1 ml aliquot of inoculum was added to each nematode-treated pot via a 20 mm deep hole made 10 mm from the base of the plant, followed by addition of 1 ml of sterile water. The hole was then filled in from surrounding soil to prevent desiccation of inoculated nematodes.

### Next-Generation Sequencing

DNA was extracted from rhizosphere samples using a PowerSoil^®^ DNA Isolation Kit (MO BIO Laboratories, Carlsberg, CA, USA). Stored soil fractions were thawed and then centrifuged at 7000 × *g* for 5 min. The supernatant was decanted and discarded. For each sample, 0.25 g of pellet was placed in a sterile 2 ml tube and the contents of one PowerSoil^®^ tube added. DNA was then extracted according to the kit manufacturer’s instructions with one exception: a 3 min mix using a bead-beater was substituted for the post-Soln C1 vortexing step. DNA extracts were quantified using a NanoDrop 2000C spectrophotometer (Wilmington, DE, USA) and then stored at -20°C for PCR reactions.

Amplification of fungal ribosomal internal transcribed spacer (ITS) sequences were performed in reaction mixtures that included 0.2 μM of each primer using iProof HF polymerase (Bio-Rad Laboratories, Hercules, CA, USA) and the manufacturers instructions. PCR cycling conditions were: 95°C for 3 min followed by 35 cycles of 95°C/30 s, 46°C/30 s, and 72°C/60 s; followed by 72°C/9 min final extension. For amplifying fungal ITS sequences, ITS3_KYO2F and ITS4R primers (Toju et al., 2012) were used with appended Illumina sequences:

ITS3_KYO2_miseqF TCGTCGGCAGCGTCAGATGTGTAT
AAGAGACAG GATGAAGAACGYAGYRAA
ITS4_miseqR GTCTCGTGGGCTCGGAGATGTGTATAAGA
GACAG TCCTCCGCTTATTGATATGC

PCR products were purified using a GeneJET PCR Purification Kit (ThermoScientific, Lithuania) and purified product was quantified using a NanoDrop 2000C spectrophotometer (Wilmington, DE, USA). ITS products were then normalized to 0.1 ng/μl using ultrapure water. For each normalized sample, 5 μl aliquots were made up to 25 μl volumes in half-skirt 96-well plates using ultrapure water containing a 467 bp exotic control sequence (synthetic zebrafish sequence appended with Illumina clamps) added to a ratio of 1:50 control sequence-to-sample DNA (i.e., 20 pg:1 ng in 25 μl volumes). Prepared plates were sent to New Zealand Genomics Limited (NZGL, Massey University, Palmerston North, New Zealand) for second PCR amplification and sequencing on an Illumina MiSeq platform.

#### Experiment 1

For each soil each treatment (clover-only control; clover with *M. hapla* added; or clover with root-feeding scarab larvae added) was replicated 12 times giving a total of 360 pots. Second instar *C. zealandica* larva were collected from soil at a site near Tokoroa (lat/long 175.90, -38.19) in the Waikato district, and stored at 4°C. A single pre-weighed larva was added to each pot (giving an equivalent of 400 larvae/m^2^) within 24 h of being collected and larvae were re-weighed at harvest when numbers of live and dead larvae were recorded. *M. hapla* inoculum consisted of 1089 (± 31 SEM) eggs and 20 (± 6) J2 juvenile *M. hapla* per ml with 346 (± 38) of the eggs being embryonated.

Clover plants were harvested after 9 weeks growth (4 weeks after *M. hapla* and *C. zealandica* inoculation). Plants were removed from pots and tipped onto a sterile surface. Soil was gently removed by hand to leave a 2 mm zone of soil around the roots (considered rhizosphere soil). *C. zealandica* larvae were removed, weighed and stored at 4°C. Total plant, shoot and root length were measured then roots were separated from stems and the stem/leaf component dried at 80°C for 48 h and dry weight recorded. All root material and associated soil was placed in a 50 ml Falcon tube with 25 ml sterile saline and vortexed at high speed for 15 s. The partially washed root material was then transferred to 20 ml fresh saline and re-vortexed for 15 s. The two soil-containing wash fractions were combined and a 0.5 ml aliquot from each sample was mixed with 60% glycerol and stored at -80°C for further analysis. The remaining soil suspension was stored at -20°C for rhizosphere DNA extraction.

Root length was recorded and the number of *Meloidogyne* galls assessed microscopically on roots in the control and nematode inoculated treatments. During the course of nematode assessment a score was assigned for degree of root rotting on a 0–3 scale with 0 being no rotting, 1 minor rotting symptoms, 2 moderate rot, and 3 severe rot including roots truncated due to root rotting. No attempt was made to determine the cause of root rots.

#### Experiment 2

Soil samples for the second trial were collected from five of the sites used in Experiment 1 (Soils 1, 4, 7, 9, and 10). Half the volume of each soil was sterilized by irradiation using 25–32 kGy followed by 15–17 kGy doses (MSD Animal Health, Upper Hutt, New Zealand). A spread plate technique using Tryptic Soy Agar and Potato Dextrose Agar (TSA and PDA) was used to test sterility of soils using a 1 g soil sample in 9 ml sterile water to prepare the dilution series and agar plates were incubated for 72 h at 20°C.

To limit macronutrient differences between irradiated and non-irradiated samples, soils were mixed with pumice. Initial moisture content was determined using 10 g samples of each soil (three replicates per soil). To determine 100% WHC of each soil: pumice mix, an equivalent of 20 g dry soil and 127 g dry pumice were weighed into pots in three replicates per soil and soaked in water for 72 h before being suspended over a tray to drain for 24 h. Final weights per replicate were averaged and used to calculate 70% WHC.

Six treatments were initiated for each soil: non-irradiated or irradiated soil, each with or without the addition of *M. hapla* or *Pyronota* (mānuka beetle) or an untreated control (*Pyronota* larvae were substituted for *C. zealandica* due to the lack of availability of *C. zealandica* at the time of the experiment. Both are root-feeding scarabs). Each treatment consisted of 10 replicates giving sixty pots per soil. The pots where *Pyronota* were added were as for the *C. zealandica* treatment in Experiment 1. The capillary mats for all sterile pots were autoclaved at 121°C for 20 min while the pots were soaked in 95% ethanol for 1 min. Each pot was filled with a mixture of 20 g dry weight equivalent soil and 127 g autoclaved 4 mm graded pumice, then watered to 70% WHC.

Seed preparation was done similarly to the first trial. The seedlings were aseptically planted, one seedling per pot, and watered with 2 ml of sterile water. Moisture of the soil:pumice mix was maintained at 70% WHC by watering every second day with sterile water for the duration of trial. All *M. hapla* egg methods were as for Experiment 1 with inoculum containing an average of 3,923 eggs (27% of eggs embryonated) and 26 J2 juveniles per ml.

*Pyronota* sp. beetle larva were collected from a site near Ohakune in the central North Island (lat/long 175.37, -39.39) on 13 November 2014, and individually weighed before aseptically adding them to pots as per *C. zealandica* in Experiment 1. Due to the late stage of larval development (L3), and to ensure sufficient larval feeding and damage to detect soil differences, two larvae were added to each pot. The larvae were added one at a time to each pot, allowing sufficient time for the first larva to bury itself into the soil/pumice mix before adding the second larva so as to minimize the possibility of larval combat. Plants were harvested during week 9 of the trial and processed and analyzed as for Experiment 1.

### Data Analyses

All plant and invertebrate data analyses were carried out in GenStat version 16. Unless otherwise stated, data were analyzed by split plot ANOVA with soils applied at whole-plot level and treatments at the subplot level. Shoot dry weight was log_e_ (n+5) transformed prior to analysis to stabilize the variance (5 is half the minimum non-zero value). *M. hapla* gall data was analyzed using split-plot REML (GenStat version 16), again with soils applied at whole-plot level and treatments at the subplot level. REML was used as the data is unbalanced with missing data for dead plants and this makes the analysis conditional on plant survival. That is, if galling had affected plant survival the means would be negatively biased. Gall data was log_e_ (n+1) transformed prior to analysis to stabilize the variance. For Experiment 1 the gall data was analyzed with root length as a co-variate. The proportion of plants without root rot (i.e., score = 0) was analyzed as split-plot using Generalized Linear Mixed Model – Binomial distribution with Logit link. The proportion of total number of plants without rot was calculated including dead plants, chosen because dead plants are likely to have had root rot (i.e., root rot is not independent of plant death).

For Illumina sequences the reads were joined using PEAR (Zhang et al., 2014) version 0.9.6 using a quality cut off of 30, a minimum joined length of 250 bp and a maximum joined length of 480 bp and a minimum overlap of 10. The sequences were assigned to the different amplicons based on exact matches to the flanking primers (minus the Illumina adapters).

The headers of the sample files were edited to add the sample name to each read. All samples were then pooled into a single file. This file was dereplicated using the Mothur (Schloss et al., 2009) program unique.seqs. In-house Ruby scripts were then applied to modify the headers of the reads to be used with the clustering program Swarm (Mahé et al., 2014). After clustering the putative OTUs were filtered based on abundance and prevalence as follows: an OTU was kept if it was present in 2% of the samples with an abundance >0.1% or in 5% of the samples with an abundance >0.01%, furthermore an OTU was kept if it represented 0.001% of the overall population in all samples together.

The representative sequence for each of the OTUs were annotated using the assign_taxonomy.py script from Qiime (Caporaso et al., 2010), where the uclust consensus taxonomy assigner was used to annotate the 16S amplicon sequences with the greengenes (McDonald et al., 2012) 13_8 database, and BLAST was used to annotate the ITS amplicon sequences with the UNITE (Kõljalg et al., 2005) 02.03.2015 release.

The OTU counts were converted into percentages of total OTU counts less the OTU corresponding to the *T. repens* reads. After treatment and other sample information was added, the OTU percentages were loaded into R (R Core Team, 2015) version 3.2.1 as tab separated values. Boxplots and *t*-test were done on subsets of the data with the default settings.

## Results

### Experiment 1

There was a wide range of pH and macronutrient levels across the soils used in this study (**Table 1**), with broad agreement between levels measured here and those by Wakelin et al. (2013) from the same soils, with the exception of pH in soil 9. The northernmost soils (1 and 2) had the greatest CEC values while soil 5 was notable for a high level of Mg and soil 8 for low total N.

**Table 1:** Macronutrient levels and pH in soils prior to sowing plants for Experiment 1 (soil number, pH and Total N data in parentheses are from Wakelin et al., 2013).

Soil number	pH	Olsen P mg/L	Sulfate S mg/kg	K mg/100 g	Ca mg/100 g	Mg mg/100 g	Na mg/100 g	Total N %	CEC mg/100 g
1 (41)	5.4 (5.7)	6	16	0.30	17.0	2.67	0.16	0.44 (0.66)	31
2 (42)	5.5 (5.5)	9	6	0.39	13.0	2.21	0.23	0.58 (0.79)	30
3 (47)	5.2 (5.2)	23	6	1.38	7.0	2.88	0.22	0.43 (0.39)	21
4 (48)	5.4 (5.6)	79	4	0.98	7.0	0.81	0.07	0.40 (0.56)	19
5 (11)	6.4 (6.8)	3	1	0.21	13.0	6.16	0.05	0.29 (0.35)	21
6 (13)	5.5 (5.4)	58	28	0.24	9.0	0.72	0.09	0.59 (0.74)	21
7 (16)	5.6 (5.3)	5	3	0.31	12.0	0.89	0.12	0.39 (0.27)	19
8 (17)	5.4 (5.2)	4	<1	0.08	3.0	0.54	<0.05	0.17 (0.19)	9
9 (37)	6.3 (5.0)	28	2	1.25	13.0	2.09	0.13	0.40 (0.39)	20
10 (27)	5.6 (5.8)	28	2	1.52	5.0	1.82	0.09	0.43 (0.49)	20

The nematodes found in the soils prior to sowing were within the ranges normally seen in pasture soil, with potentially plant damaging populations of *Pratylenchus* present in soil 3 in Experiment 1 and of *Heterodera* and *Pratylenchus* in soil 7 for Experiment 2. Sequencing of the ITS region of *Meloidogyne* observed in soil extracts prior to sowing (**Table 2**) showed the presence of *M. trifoliophila* in soil 3 and *Meloidogyne fallax* in soil 4. No usable sequence information was obtained from *Meloidogyne* specimens in soil 2, but morphological observations of root galls from clovers growing in the soil at field sampling and of J2 juveniles in the soil extract prior to sowing suggest these were also *M. trifoliophila*. None of the populations of other plant feeding nematode genera were likely to be having a significant effect on clover growth, with the possible exception of the *Pratylenchus* population in soil 3. In addition to the plant feeding nematodes listed in **Table 2**, Criconematidae were observed from soil 10 and *Tylenchorhynchus* from soil 9 (both 10/100 g dry soil).

**Table 2:** Total nematodes and plant feeding genera per 100 g dry soil and soil moisture of subsamples after sieving and prior to being used to fill pots.

Soil number	Total nematodes	*Meloidogyne*	*Heterodera*	*Pratylenchus*	*Helicotylenchus*	*Paratylenchus*	Soil moisture (%)
**Experiment 1**							
1	644	0	10	0	8	0	30
2	494	11	3	0	31	17	39
3	1508	46	7	459	0	14	16
4	966	13	0	0	0	93	11
5	493	0	11	0	56	0	12
6	1835	0	6	17	188	0	38
7	2164	0	0	33	0	131	17
8	805	0	0	59	208	59	13
9	1576	0	0	22	182	27	11
10	5227	0	9	94	99	260	18
**Experiment 2**							
1	2957	74	0	0	92	0	85
4	1359	0	115	124	0	53	33
7	1054	0	290	145	0	0	32
9	3394	0	0	293	0	829	28
10	2096	13	238	75	0	213	26

**FIGURE 3:**
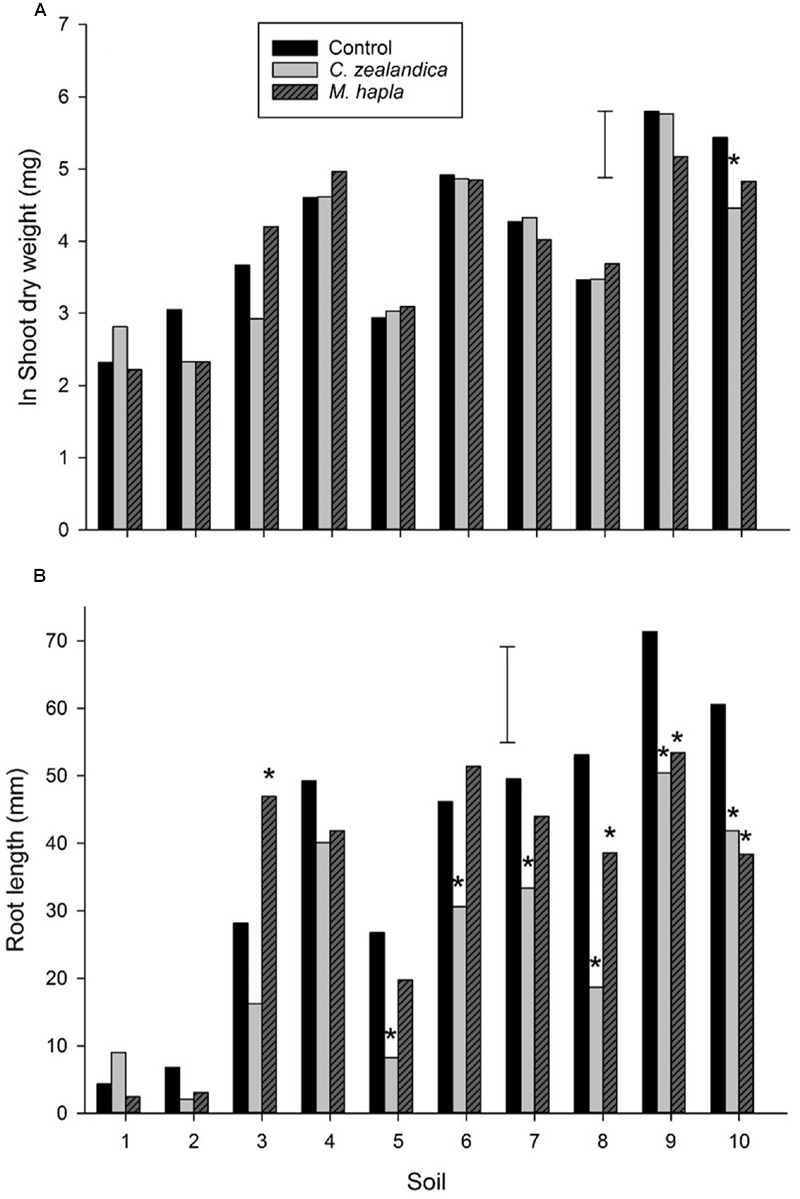
**Experiment 1: Growth of white clover shoots **(A)** and roots (B)**, without invertebrate pests (control) or inoculated with *Costelytra zealandica* or *Meloidogyne hapla.* Error bars are lsd 5%. Soils are arranged from most northerly (1) to most southerly (10). Astrices denote significant difference to control within each soil.

There was a large range of shoot and root growth across the soils (**Figure 3**) which illustrates the range of white clover productivity across New Zealand. There was no clear pattern for either soil type (brown or recent) or grazing system (dairy or sheep and beef) as drivers for these growth differences. Geographically, there was a tendency for lower shoot and root production in more northerly soils, with the greatest production from the southernmost soils. With temperature and moisture not limiting factors in these pot trials other factors such as soil nutrients and pest invertebrates (e.g., nematodes) and diseases were likely drivers of growth differences. Unsurprisingly, there were positive correlations between soil P and K and clover shoot and root growth but these were not significant. There was a significant negative correlation between soil CEC and root length (*r* = -0.736, *P* < 0.05) (**Table 1**; **Figure 3**).

Addition of *C. zealandica* larva or *M. hapla* eggs to pots significantly reduced clover shoot weight in only one of the soils (**Figure 3**). However, *C. zealandica* feeding significantly reduced root length compared to control plants in all the South Island soils. It is likely that the mesh inserted in the *C. zealandica* pots ensured there was sufficient root system remaining to support similar shoot growth to the control plants. There were some significant differences in the weight of *C. zealandica* initially added to soils (**Table 3**). Soils 5 and 8 were inoculated with larvae that were significantly heavier than those in soils 4 and 7. However, those differences had disappeared by the time of harvest. Significantly fewer grubs survived the 4 week duration between inoculation and harvest of the experiment in soils 4 and 10 than most other soils.

**Table 3:** Experiment 1: Mean *Meloidogyne* nematode root galls (back-transformed data with log_e_-transformed data in parentheses) and weight of *C. zealandica* larvae at inoculation into pots (Initial) and at harvest (Final).

	Number galls		*C. zealandica*			
Soil number	Control	Nematode inoculated	Initial weight mg	Final weight mg	Weight change mg	No. alive/pot
1	0.1 (0.13)	4.7 (1.74)^∗^	31.3	37.3	5.9	0.92
2	0.5 (0.38)	2.2 (1.15)^∗^	30.4	45.1	12.9	0.84
3	5.0 (1.80)	11.6 (2.53)^∗^	31.3	35.4	5.7	0.83
4	0.0 (0.03)	2.8 (1.33)^∗^	28.5	40.1	11.1	0.45*
5	1.2 (0.81)	2.9 (1.37)	34.4	43.7	9.0	0.71
6	0.0 (0.01)	7.2 (2.10)^∗^	30.7	40.4	9.7	0.76
7	0.0 (-0.02)	0.7 (0.52)	28.3	46.8	16.6	0.62
8	1.5 (0.93)	6.1 (1.96)^∗^	34.1	39.7	3.8	0.72
9	0.0 (-0.02)	0.6 (0.48)	30.0	46.9	16.2	0.93
10	0.0 (-0.01)	6.2 (1.97)^∗^	33.9	47.8	11.7	0.30*
Lsd 5% _soil_	—		4.95	13.10	9.24	0.348
Lsd 5% _treatment^∗^soil_	(0.647)		—		–	–

*Lsd values are to compare: across soils (_soil_) or treatments across soils (_treatment∗soil_). Astrices denote significant difference to control for nematode data and significant difference to the soil with the greatest survival rate for *C. zealandica*.*

Inoculation with *M. hapla* did not result in significant changes in clover shoot growth in any of the soils (**Figure 3**). However, it is notable that the only soils where *M. hapla* inoculation resulted in significant reductions in root length were from the southern South Island where clover-feeding *Meloidogyne* have until recently only been rarely encountered in pastures (personal observation).

**FIGURE 4:**
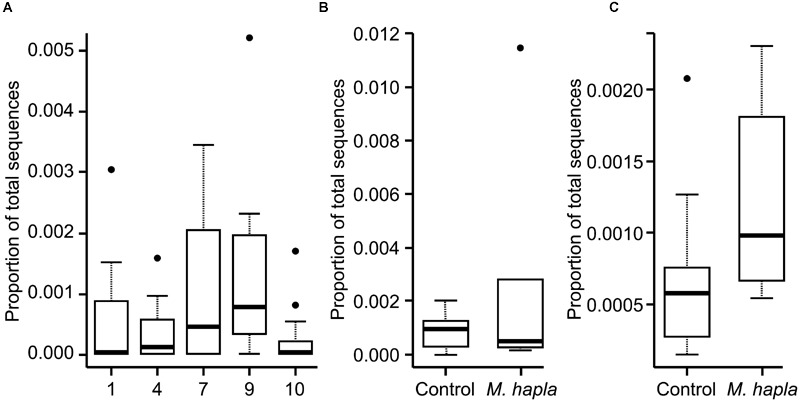
**Proportion of all fungal ITS DNA sequences from Experiment 1 that belonged to Orbiliomycetes for: (A)** all soils; **(B)** soil 7 with or without added *M. hapla* and **(C)** soil 9 with or without added *M. hapla*.

Inoculation with *M. hapla* significantly increased root galling in all but soils 5, 7, and 9 (**Table 3**). It is not clear what was responsible for that effect in soil 5 but in soils 7 and 9 fungal community analysis by high-throughput ITS sequencing showed that there were significantly greater abundances of Orbiliomycetes fungal sequences in those than in other soils. Addition of *M. hapla* further increased abundance of these fungi (**Figure 4**).

For root rotting score there was no significant difference between the control and *M. hapla* inoculated soil so results were pooled. Mean root rot scores were generally low to minor severity (**Table 4**). There were significant differences in root rotting incidence between soils, with soils 1 and 10 having the lowest proportion of plants without any signs of rot and this was significantly different to all other soils.

**Table 4:** Experiment 1: Arithmetic mean root rot score and back-transformed proportion of control roots without rot (Logit transformed data ± SEM in parentheses) for Experiment 1.

Soil number	Root rot score (0–3 scale)	Proportion of roots without rot
1	1.1	0.12 (-2.00 ± 0.68)
2	0.2	0.46 (-0.17 ± 0.43)
3	0.3	0.71 (0.90 ± 0.47)
4	0.2	0.71 (0.90 ± 0.47)
5	0.3	0.50 (0.00 ± 0.49)
6	0.6	0.55 (0.20 ± 0.47)
7	0.2	0.82 (1.55 ± 0.63)
8	0.1	0.82 (1.55 ± 0.63)
9	0.0	0.88 (2.00 ± 0.68)
10	1.0	0.27 (-0.97 ± 0.51)

### Experiment 2

A second trial was set up to validate the results from Experiment 1 and to directly test the impact of the overall soil microbial content by using a pumice/soil mixture to reduce abiotic effects and comparing biotic with sterilized soil. A different scarab beetle was also used to measure insect effects – in this case a *Pyronota* sp. scarab due to the lack of available *C. zealandica* larvae.

From extractions carried out before sowing clover there was a greater abundance of total nematodes in all but soil 10 compared to the samples collected for Experiment 1 (**Table 2**). These differences in initial nematode population numbers and plant feeding genera present between Experiments 1 and 2 reflect differences in the time of year sampling was carried out and concomitant greater soil moistures. *Heterodera* and *Pratylenchus* spp. were observed at moderate to high populations in all but soil 1. *Meloidogyne* spp. were observed in two soils, neither of which had this genus present in samples taken for Experiment 1. Morphology of J2 juvenile tails and galls suggested the *Meloidogyne* from soil 1 were *M. trifoliophila.* The *Meloidogyne* sp. present in soil 10 was identified from tail morphology as the grass-feeding *M. naasi*. Some of the individuals observed were heavily encumbered by the nematode-pathogenic bacteria *Pasteuria* sp.

The pumice used to dilute soils had notably greater pH and Na levels than any of the soils used in this experiment, so that these parameters were elevated in the soil:pumice mix used (**Table 5**). Irradiation produced no consistent differences in macronutrient levels in the pumice: soil mix. The largest changes due to irradiation were in Olsen P in soils 1, 4 and 10; K in soils 9 and 10; and Ca in soil 1. pH was generally increased after irradiation (by 0.1–0.3), except for soil 10 where no change in pH was observed. Compared to soils alone used in Experiment 1 (**Table 1**) the soil:pumice mix used in Experiment 2 resulted in a reduction in the magnitude of differences in the pH and macronutrient parameters amongst the five soils. Spread plating of irradiated soils showed no microbial growth.

**Table 5:** Macronutrient levels and pH in pumice alone, soil alone or soil:pumice mix (Mix) prior to sowing plants for Experiment 2.

Soil number	Soil treatment	pH	Olsen P mg/L	Sulfate S mg/kg	K mg/100 g	Ca mg/100 g	Mg mg/100 g	Na mg/100 g	Total N %	CEC mg/100 g
Pumice alone	—	7.7	3	<1	0.58	2.3	0.57	0.28	<0.04	4
1 Soil alone	—	5.7	6	11	0.59	19.1	3.61	0.16	0.49	36
1 Mix	—	6.3	5	2	0.77	4.6	1.01	0.30	0.06	9
1 Mix	Irradiated	6.6	11	4	0.64	5.9	1.08	0.26	0.07	9
4 Soil alone	—	5.6	65	7	0.57	6.9	0.99	0.08	0.41	19
4 Mix	—	6.0	26	2	0.57	3.4	0.67	0.24	0.08	7
4 Mix	Irradiated	6.3	19	2	0.62	3.2	0.57	0.29	0.08	6
7 Soil alone	—	5.4	5	5	0.32	9.0	0.87	0.09	0.37	19
7 Mix	—	6.0	3	1	0.40	3.6	0.65	0.16	0.10	7
7 Mix	Irradiated	6.2	5	1	0.46	3.2	0.64	0.22	0.10	6
9 Soil alone	—	5.6	15	2	0.58	6.4	1.19	0.07	0.30	14
9 Mix	—	6.5	4	1	0.66	2.9	0.64	0.30	0.08	5
9 Mix	Irradiated	6.6	7	1	0.47	2.8	0.61	0.19	0.08	5
10 Soil alone	—	5.5	10	3	1.07	3.1	1.26	0.06	0.24	17
10 Mix	—	6.0	4	2	0.68	2.7	0.70	0.32	0.08	7
10 Mix	Irradiated	6.0	10	2	0.81	3.1	0.77	0.31	0.08	7

**FIGURE 5:**
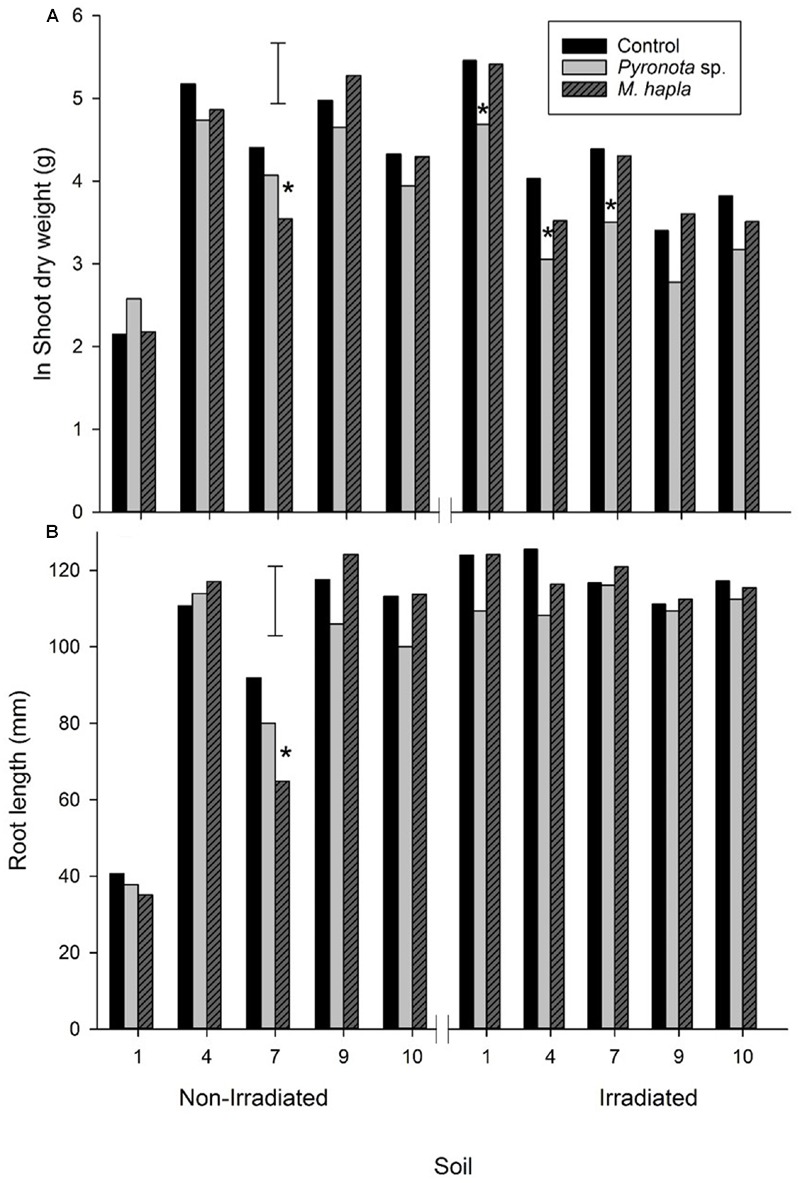
**Experiment 2: Growth of white clover shoots **(A)** and roots (B)**, without invertebrate pests (control) or inoculated with *C. zealandica* or *M. hapla*, either before or after soil irradiation. Error bars are lsd 5%. Soils arranged as for **Figure 3**. Astrices denote significant difference to control within each soil.

For all soils combined there was a significant reduction in clover shoot weight in irradiated (ln shoot weight = 3.94 g) compared to non-irradiated (4.12 g) soil (lsd 5% = 0.179 g). For control soils irradiation significantly reduced shoot growth in soils 4 and 9 but significantly increased white clover shoot growth in soil 1 and resulted in no significant difference in the remaining two soils (**Figure 5**). Changes in shoot weight in irradiated vs. non-irradiated soil for invertebrate treatments largely mirrored those for the control with the exception of the nematode treatment in soil 7 where plants in irradiated soil produced significantly greater shoot mass than those in non-irradiated soil.

For all non-irradiated soils combined there was no significant effect of invertebrate treatment on shoot growth. However, in irradiated soils there was a significant decrease in white clover shoot dry weight in the mānuka beetle treatment (ln shoot weight = 3.49 g) compared to the control (4.24 g) and *M. hapla* (4.10 g) treatments (lsd 5% = 0.309) (**Figure 5**). For individual soils the only significant decreases in shoot growth compared to control occurred in *M. hapla* treatment in non-irradiated soil 7 and in the mānuka beetle treatment in irradiated soils 1, 4, and 7.

Irradiation significantly increased root length over all soils combined (116.0 mm) compared to non-irradiated soil (91.1 mm, lsd 5% = 4.71), which was largely due to the increases in root length due to irradiation of soils 1 and 7 (**Figure 5**). Mānuka beetle significantly reduced root length for all soils combined (99.3 mm), compared to the control (106.9 mm) but this was not the case with the *M. hapla* treatment (104.5 mm, lsd 5% = 5.77), with no interaction due to irradiation (*P* = 0.825). The only significant difference in root length, compared to control soil, due to invertebrate pests for individual soils was in non-irradiated soil 7 where the *M. hapla* treatment gave a decrease in root length. It is possible the combined populations of *Heterodera* and *Pratylenchus* in soil 7 (**Table 2**) had an impact on clover root and shoot growth, in addition to the added *M. hapla*.

For soils 4 and 9 inoculation of plants with *M. hapla* resulted in significantly more nematode galls in both irradiated and non-irradiated soil, compared with the non-irradiated control soil (**Table 6**). In soils 1, 7, and 10 only inoculation into irradiated soil resulted in increased galling. In contrast to the other two soils with this effect, the addition of *M. hapla* to soil 7 also increased root rotting (**Table 7**), and both clover root and shoot growth were significantly reduced in this treatment (**Figure 5**).

**Table 6:** Experiment 2*:* Mean number of *Meloidogyne* root galls and weight of *Pyronota* larvae at inoculation into pots (Initial) and at harvest (Final).

	Number galls		*Pyronota* sp.	
Soil number	Control	*M. hapla* inoculated	Initial weight (mg)	Final weight (mg)	Weight change (mg)	No. alive/ pot
1	8.9	7.9	62.2	43.0	-19.2	0.9^∗^
4	-0.9	7.3*	62.1	46.9	-15.2	1.5
7	0.0	4.6	62.4	44.6	-17.7	1.7
9	-1.0	9.3*	63.2	41.2	-22.0	1.5
10	0.2	5.6	62.6	48.8	-13.7	1.5
1 Irradiated		19.6*	61.9	52.4	-9.3	1.3
4 Irradiated		6.5*	61.8	57.3	-4.3	1.2
7 Irradiated		11.9*	62.3	41.3	-21.1	1.2
9 Irradiated		8.2*	62.6	44.8	-17.8	1.5
10 Irradiated		14.7*	62.0	49.0	-13.0	1.7
Lsd 5%	6.41		1.57	11.11	11.15	0.61

*Astrices denote significant difference to control for nematode data and significant difference to the soil with the greatest survival rate for *Pyronota*.*

**Table 7:** Experiment 2*:* Arithmetic mean root rot score and nodules/plant.

Soil number	Root rot score (0–3 scale)		Rhizobial nodules/mm root	
	Control	*M. hapla* inoculated	Control	*M. hapla* inoculated
1	2.1	2.2	0.16	0.15
4	0.3	0.8	0.22	0.18
7	1.1	1.7^∗^	0.17	0.14
9	0.0	0.0	0.16	0.16
10	0.1	0.5	0.09	0.11
1 Irradiated		0.0^∗^		0.10
4 Irradiated		0.0		0.03^∗^
7 Irradiated		0.0^∗^		0.02^∗^
9 Irradiated		0.0		0.07^∗^
10 Irradiated		0.0		0.02^∗^
Lsd 5% (treatment^∗^soil)	0.51		0.065	

*Astrices denote significant difference to control.*

**FIGURE 6:**
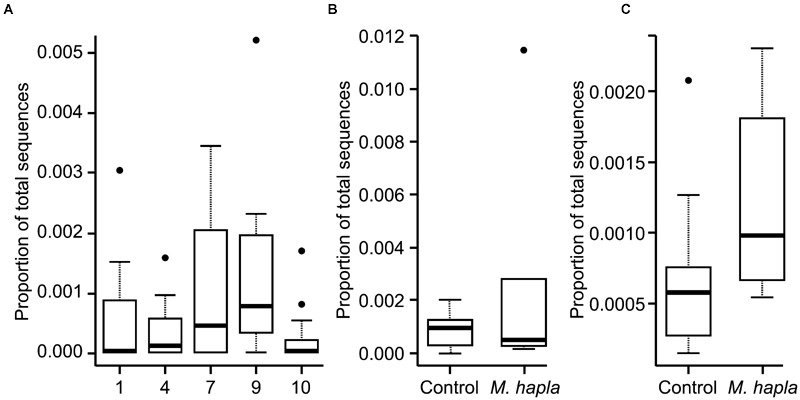
**Proportion of all fungal ITS DNA sequences from Experiment 2 that belonged to Orbiliomycetes for: (A)** all soils; **(B)** soil 7 with or without added *M. hapla* and **(C)** soil 9 with or without added *M. hapla*.

The proportion of the sequences that were of Orbiliomycetes fungi was much lower in this experiment that in the previous one (**Figure 6**), with their proportion approximately in line with the soil dilution rate (ca. 6×) for soils 7 and 9 but much lower than that in soil 4. As for Experiment 1 these abundances increased in response to added *M. hapla* but in this case none of the increases were significant.

The initial weights of *Pyronota* larvae added to pots was not significantly different between soils or treatments (data not shown), however final weights and, therefore weight change of larvae at the end of the experiment were significantly different amongst some soils and treatments (**Table 6**). In soils 1, 4, and 9 there was an increase in final weight of larvae in irradiated vs. non-irradiated soil, which was significantly so for soil 4. Significantly less beetle larvae survived in non-irradiated soil 1 (0.9) compared to non-irradiated soil 7 (1.7, lsd 5% = 0.61) but there was no significant difference in beetle survival amongst irradiated soils. Some larvae had developed through to pupae and adults at the end of the experiment and were discounted from the analysis of final weights.

Irradiation eliminated root rot from all soils (mean score = 0.0) which was significantly less than the overall score in control (0.8) which in turn was significantly less than in nematode treated soil (1.0, lsd 5% = 0.2). This effect was significant for soil 7. Soil 9 was the only one of the five soils in which root rot was not observed in any of the treatments (**Table 7**). Similar to root rot the number of rhizobial nodules was significantly reduced in irradiated soil inoculated with *M. hapla* (0.05/ mm root) compared to control (0.16/mm) and *M. hapla* treated non-irradiated soil (0.15, lsd 5% = 0.029). The only individual soil for which this did not hold true was soil 1 where nodules/ mm root in irradiated soil inoculated with *M. hapla* was not significantly different to control or *M. hapla*-inoculated non-irradiated soil (**Table 7**).

## Discussion

Invertebrate pests had deleterious effects on *T. repens* root and/or shoot growth in some soils, across both experiments. In a minority of soils, however, invertebrate pests had no significant impacts on plant growth or damage in either experiment. For *M. hapla* it appears Orbiliomycetes fungi were responsible for this effect. The *M. hapla* suppressive effects observed in soils 7 and 9 are, to our knowledge, the first report of nematode suppressive soils from New Zealand. Certainly there has been no reported *in situ* association between Orbiliomycetes fungi and *Meloidogyne* in this country, and few worldwide (Singh et al., 2012). Orbiliomycetes fungi are a monophyletic group which include the genera *Arthrobotrys, Drechslerella*, and *Dactylellina* (Li et al., 2005). The use of culture independent next generation sequencing in this study has enabled the relationship between these fungi and a damaging plant-feeding pest to be revealed much more readily than would have been possible with culture-dependant techniques.

Although, many studies have tried to isolate and utilize nematode-trapping fungi as biocontrol agents for plant-feeding nematodes, they have yet to be utilized commercially (Degenkolb and Vilcinskas, 2016). This may be because the fungi that have thus far been worked with have been selected mainly on their ability to be readily cultured which is clearly an important determinant for any potential marketable biocontrol agent. However, because these fungi are often facultative parasites such an approach may overlook those fungi that are actually reacting to the nematodes of interest, and this is an area where next generation sequencing, as shown in this study, can be a useful guide to selection of potential biocontrol agents for specific nematodes. Additionally, the results presented here support the need for future work directed at the use of Orbiliomycetes as bioindicators for nematode suppressive soils. Such bioindicators could be used to inform the decision making process of land managers contemplating actions which could disrupt existing biocontrol mechanisms (e.g., soil cultivation). As mentioned earlier a substantial comparison of the high resolution microbial communities will form the basis of a separate paper.

For soil 7 irradiation removed the nematode-suppressive effect. Although addition of *M. hapla* to pots of this soil did not significantly increase root galling in either experiment it did significantly increase root rotting and this increase was associated with significantly reduced root length. It is possible the invasion of added *M. hapla* into roots created entry points for disease organisms. Once microbes were eliminated by irradiation a significant increase in galling was observed, likely due to elimination of Orbiliomycetes fungi but this was not associated with a decrease in root length. It is possible the impact of eliminating root pathogens was greater than that of the relatively small amount of galling caused by nematodes. White clover is a very good host for the resident *M. trifoliophila* in soil 3 (Mercer and Miller, 1997), as illustrated in this case by the relatively large amount of galling observed on plant roots from the control treatment. The *M. fallax* observed in soil 4 prior to sowing is also hosted by white clover (Rohan et al., 2016) but in this case appears to have been present in too few numbers initially to be able to establish a population causing noticeable galling in the control treatment, as was also the case for the putative *M. trifoliophila* in soil 2.

Along with the resident and added invertebrates, the microbial flora of soils can have a profound impact on plant growth and in this study positive, negative, and neutral soil microbiota impacts on *T. repens* were all observed. Clearly, irradiation had a considerable impact on white clover growth in soil 1, significantly increasing both root and shoot growth. It appears pathogenic microbes in that soil were having a large impact on potential productivity with this soil having the greatest root rot scores of any of the soils. There have been few studies of the causes of root rotting in New Zealand pasture plants. The soils with the lowest proportion of rot-free plant roots were from Northland (soil 1) and Southland (soil 10), with root rot incidence likely helping to explain the poor growth of plants in soil 1. Skipp and Christensen (1983) recorded the incidence of potential root-rotting fungi on white clover roots during a survey of New Zealand pastures and found that *Codinea fertilis* predominated in root pieces from two soils in Northland (close to soil 1 in the current study) and that *Bimuria novae-zelandiae* predominated in soils of Southland (close to the current soil 10). Wakelin et al. (2016) found a significant negative association between a *Pythium* clade and *T. repens* shoot growth. Whether, these are the causative agents of the root rotting observed in the current study will be clarified by the next generation sequencing results to be carried out on white clover endosphere samples which will be reported in a future paper.

Other than soil 1 irradiation either significantly reduced (soils 4 and 9) or had no significant effect (soils 7 and 10) on shoot growth for the control treatments. It appears these effects are due to changes in the microbial flora such that in soils 4 and 9 elimination of predominant beneficial microbes reduced plant growth whereas irradiation impacts on both beneficial and deleterious microbes in soils 7 and 10 resulted in no change in plant growth. Changes in soil nutrient levels post-irradiation are not able to explain the plant growth effect observed because they are not consistently associated with either increases or decreases in plant growth. At the rates of irradiation used here (ca. 40 kGy) Buchan et al. (2012) observed a decrease in NO_3_-N (from ca. 4 to 0 μg N/g dry soil) and an increase of NH_4_-N (from 1 to 8–12 μg) compared to untreated soil, probably due to the elimination of nitrifying bacteria. They also observed that elimination of microbes by irradiation increased pools of labile carbon. It had been expected that these changes would occur in the soils used here and that these would affect plant growth consistently across all soils (NH_4_ driving either an increase or decrease in *T. repens* growth depending on its concentration in soil (Britto and Kronzucker, 2002). However, this did not occur which suggests that any effects that occurred due to changes in nutrient status were, at least to some extent, overridden by changes in microbial communities and their effect (positive or negative) on plant growth. The soil dilution method used in Experiment 2 would have minimized differences in nutrients due to irradiation, compared to non-irradiated controls.

Wakelin et al. (2016) used microwaving to sterilize a range of New Zealand soils and found that of 10 soils they tested all but one gave a significant positive response in *T. repens* growth in sterilized soil. Over all soils they found that control soil produced 28.6% more *T. repens* shoot weight than sterile soils. Included in their soil set was soil 9 which, with their sterilization technique, showed a *T. repens* growth increase of ca. 25% in response to soil sterilization. This contrasts with the >200% reduction in growth in irradiated compared to untreated from the current study. There are likely a range of reasons for this difference between studies including temporal (the soils were sampled in different years), spatial (diseases, for instance, can be patchily distributed) and the severity of the treatment imposed. In the current study virtually all microbes were eliminated while the Wakelin et al. (2016) method was less severe and may have retained some beneficial microbes which then minimized the negative effects of the sterilization treatment. Root length in all soils were greater in Experiment 2 than Experiment 1, with many root systems reaching the bounds of the pot depth (120 mm). Dilution of the field soil with pumice appears, then, to have had one of the desired effects of reducing the loading, and therefore impact, of root pathogens or other deleterious microbes.

Although, survival of *C. zealandica* in soil 10 was very low this was not reflected in a significantly lower weight gain of those grubs that did survive, suggesting that quality of the food resource was not the cause of the grub’s demise. Indeed, there was a significant reduction in root length of plants growing in soil 10 with grubs which indicates the grubs survived sufficiently long to eat a large proportion of roots but subsequently succumbed. Similarly, there was lower survival of grubs in soil 4 than in many other soils but in this case there was no significant reduction in root length, suggesting grubs may have succumbed more rapidly than in soil 10. Grubs in soil 4 were significantly smaller than those in soil 10 at inoculation to pots which may help explain why they succumbed more rapidly to whatever was the cause of death of these grubs. There was no correlation between *C. zealandica* and any of the fungi sequenced (data not shown) so it is likely these effects are due to other microbes such as the bacteria *Serratia entomophila* which is known to cause disease in these insects (Hurst et al., 2000). There was no similar effect of these two soils on *Pyronota* sp. survival in the second trial, suggesting the host specificity of whatever microbe was responsible for *C. zealandica* deaths did not extend to *Pyronota*. *Pyronota* sp. had no significant impact on either roots or shoots in any of the non-irradiated soils but did significantly decrease shoot growth in soils 1, 4, and 7 post-irradiation suggesting that any control exerted within control soils was eliminated by irradiation.

This study has clearly shown the effects of soil biology on plant growth can be profound. The *in situ* interactions between soil microflora and fauna that occur in the rhizosphere deserve closer attention, especially in New Zealand soils where they are little studied. Insights that can be gained from such studies will form the basis for developments in biocontrol, bioindicators and the agronomic practices that support beneficial interactions.

## Author Contributions

NB, DF, and RDJ conceived the project and gained grant funding. NB, KA, RJ, RDJ, YM, GB, AP, and DF had substantial contribution to the acquisition and interpretation of data. VC, CC, and PM had substantial contribution to data analysis and interpretation. All authors had substantial contributions to the drafting, revising, final approval, and agreement on the manuscript.

## Conflict of Interest Statement

The authors declare that the research was conducted in the absence of any commercial or financial relationships that could be construed as a potential conflict of interest.
